# Spatial cytotoxic and memory T cells in tumor predict superior survival outcomes in patients with high‐grade serous ovarian cancer

**DOI:** 10.1002/cam4.3942

**Published:** 2021-05-05

**Authors:** Guodong Zhang, Qing Xu, Xiangyun Zhang, Moran Yang, Yiying Wang, Mengdi He, Jiaqi Lu, Haiou Liu

**Affiliations:** ^1^ Obstetrics and Gynecology Hospital Fudan University Shanghai China; ^2^ Department of Obstetrics and Gynecology of Shanghai Medical School Fudan University Shanghai China; ^3^ Department of Gynecology Suzhou Municipal Hospital Suzhou China; ^4^ Shanghai Key Laboratory of Female Reproductive Endocrine Related Diseases Shanghai China; ^5^ Department of Gynecology Kashgar Prefecture Second People’s Hospital Kashi China

**Keywords:** cytotoxic T cells, high‐grade serous ovarian cancer, memory T Cells, overall survival, prognostic model

## Abstract

Although the association between tumor‐infiltrating CD3^+^ T and CD8^+^ T cells and superior survival in high‐grade serous ovarian cancer (HGSOC) has been observed, the different spatial localization of tumor‐infiltrating lymphocytes (TILs) possesses heterogeneous effects. We performed localized measurements in 260 HGSOC from 2 independent cohorts represented in tissue microarray format to determine the localized expression pattern and clinical significance of CD3^+^ T, CD8^+^ T, and CD45RO^+^ cells in HGSOC. Different density of spatial localization of CD3^+^ T, CD8^+^ T, and CD45RO^+^ cells exhibited heterogeneous association with OS. The combination of the center of the tumor and invasive margin localized CD8^+^T cells (CD8^CT&IM^) with the same margin localized CD45RO (CD45RO^CT&IM^) was the most robust prognostic predictor. Immune score (IS) was constructed by integrating FIGO stage with CD8^CT&IM^ and CD45RO^IM&CT^ and had the best prognostic value in HGSOC. The low‐, intermediate‐, and high‐IS groups were observed in 44.7%, 41.6%, and 13.7% of patients, respectively. Low‐IS identified patients were at higher risk of death compared to high‐IS identified patients (HR = 12.426; 95% CI 5.317–29.039, *p* < 0.001); meanwhile, we evaluate the RMSTs over 10 years of follow‐up and obtained RMST values of 104.09 months (95% CI 96.31–111.87 months) in the high‐IS group, 75.26 months (95% CI 59.92–90.60 months) in the intermediate‐IS group, and 48.68 months (95%CI 38.82–58.54 months) in the low‐IS group. In general, spatial localization can modulate the clinical effects of TILs in HGSOC. Thus, the spatial expression of CD8 and CD45RO could aid clinicians to determine the follow‐up plan of patients with HGSOC.

## Graphical abstract

Infiltrating T cells in distinct areas associated with different clinical outcomes, and cytotoxic and memory T cells combing clinical characteristic can efficiently predict survival in clinical practice.

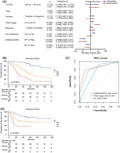

## 1 INTRODUCTION

Epithelial ovarian tumor is the seventh most common female cancer and the overall 5‐year survival rate is only 46%.^1^ The clinical manifestations of epithelial ovarian tumors are not obvious in the early stage, and about 75% of patients have entered the advanced stage at the time of diagnosis.^1^, ^2^ High‐grade serous cancer (HGSOC) has the highest incidence and mortality among different types of epithelial ovarian cancer.^3^ In recent years, the survival rate of patients with HGSOC has hardly been improved because of early detection, relapse, drug resistance, and other characteristics.^4^

Previous studies have demonstrated that heterogeneity of immune cell infiltration in ovarian cancer which may influence the development, occurrence, and prognosis of ovarian cancer.^5^, ^6^, ^7^, ^8^, ^9^ T cells have been found to play an important role in the treatment of tumors, and CD3^+^, CD8^+^, and CD45RO^+^ T cells have been individually reported associated with favorable survival.^10^, ^11^, ^12^ Several studies have observed that the combination of CD8^+^ and CD45RO^+^ T cells was a better indicator of patient prognosis in colorectal cancer.^13^, ^14^, ^15^, ^16^ In addition to the quantity, the location of immune cell infiltration has a different contribution to prognosis^11^, ^17^, ^18^, ^19^

In this study, we confirmed that infiltrating intertumoral T cells were correlated with a decreased incidence of death. Then, the location of CD3^+^, CD8^+^, and CD45RO^+^ T cells was also correlated with overall survival and the combination of them has more significance. Additionally, a prognostic model of the T cells and clinical information was established and validated.

## 2 MATERIALS AND METHODS

### 2.1 Population

Training Cohort: A retrospective series of 190 formalin‐fixed paraffin‐embedded (FFPE) samples were obtained from patients with HGSOC who underwent primary surgery in the absence of neoadjuvant chemotherapy between March 2013 and November 2015 from Obstetrics and Gynecology Hospital of Fudan University. All samples were taken from primary lesions. Pathology staging was performed according to the FIGO (International Federation of Gynecology and Obstetrics) classification (2018), and histologic types were determined according to the current WHO classification.^20^ Data on clinical outcomes were obtained retrospectively by interrogation of families and the last follow‐up is September 2019. Validation Cohort: A retrospective series of 70 formalin‐fixed paraffin‐embedded samples from patients of HGSOC was collected between May 2013 and August 2018 at Suzhou Municipal Hospital who underwent primary surgery in the absence of neoadjuvant chemotherapy. All samples were taken from primary lesions. Data on clinical outcomes were obtained retrospectively by interrogation of families and the last follow‐up is March 2019. The clinicopathological characteristics of the training and validation cohort were both shown in Table S1. There was no difference of all clinicopathologic characteristics between the two cohorts but ascites. Perhaps it is because the limited patients and different areas. All participants included in this study provided written informed consent, which allowed us to use their specimens and data for publication. The objective assessment of progressive disease was determined by central radiologic and clinical review in a blinded manner, according to RECIST (Response Evaluation Criteria in Solid Tumors), version 1.1.^21^

Patients were divided to the low CA125 concentration group with a concentration equal to or below 35 U/ml, while the high CA125 concentration group possessed concentrations higher than 35 U/ml.^22^ The LN metastasis was defined the patients with clinical and pathological diagnosis of LN metastasis except patients unreceived cytoreductive surgery which did not need pathological diagnosis.^23^

### 2.2 Tissue microarray construction and immunohistochemistry

**FIGURE 1:**
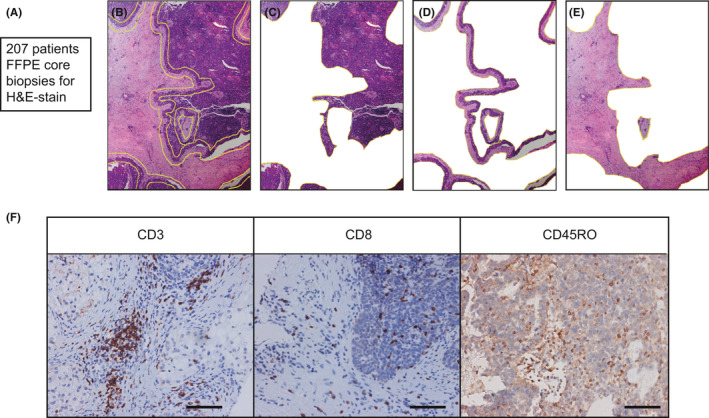
Examination of CD3^+^ T cells, CD8^+^ cytotoxic T cells, and CD45RO^+^ memory T cells in epithelial ovarian cancer. A‐E. Representative H&E–stained slides of epithelial ovarian cancer tissues. (B) were evaluated for the presence of the CT (C), IM (D), and IN (E). F. Representative examples of CD3, CD8, and CD45RO immunostaining (brown) of epithelial ovarian cancer tissue microarray. Bar=100 μM,IHC

Hematoxylin and eosin (H&E)–stained slides of tissues were reviewed without knowledge of clinical characteristics or outcomes. Each section H&E‐stained slide was evaluated for the presence of the center of the tumor (CT), the invasive margin (IM), and interstitium (IN) (Figure 1A‐E).^13^, ^17^ For each tumor specimen, two 4‐μm cores containing regions of CT, IM, and IN were selected were taken from the areas containing the highest density of immune cells to make tissue microarray. Immunohistochemistry (IHC) was performed through deparaffinizing, rehydrating, antigen retrieval, blocking, incubating primary and secondary antibody, visualizing with applicable antibodies CD45RO (OPD4), CD8 (4B11), and CD3 (SP7).

To assess the density of stained immune cells, three representative areas were screened under a 200x microscope vision by two separate pathologists blinded to patient clinicopathological data and clinical results. IHC staining cells in each region were quantified and counted as the number of cells in each region. The two pathologists independently scored the microarrays and paired the score at the end. The microarrays were scored independently by two pathologists who matched scores at the end. The average of their evaluations was used to count. The count of variation, more than 3 cells, was reassessed by two pathologists to gain consensus. The coefficients of variation between two different cores and two separate pathologists were both <15%. Meanwhile, an algorithm developed for the NIH software ImageJ was also used to make sure the repeatability of results. The range for IHC score was from 0 to 38. The cut‐off value for basophils high/low subgroups was determined by X‐tile software (X‐tile Version 3.6.1, Yale University, CT, USA, https://xtile. software.informer.com/). The number 0 was selected as the cut‐off and the cohort was divided into immune cell negative (neg) and positive (pos) groups. In addition, we divided the cohort into double positive (DP), single positive (SP) and negative groups when two kind of immune cells used.

### 2.3 Statistical analysis

Kaplan–Meier curves and Cox proportional hazards regression were performed by Medcalc software (version 18) and their significance was assessed by the log‐rank test. The receiver operating characteristic (ROC) curves, area under curve (AUC), Akaike information criterion (AIC), index of concordance (C‐index), and the restricted mean survival time (RMST) were performed R software packages (version 3.5.1). A chi‐square test was used to examine the significance between different immune cell infiltration groups and clinical information by Medcalc software (version 18).

## 3 RESULTS

### 3.1 Density and location of T Cells associated with prognosis in HGSOC

In order to study the infiltration of T cells, we investigated the densities of CD3^+^, CD8^+^, and CD45RO^+^ T cells infiltration in the mass (All) and regions (CT, IM, and IN) which staining brown by IHC and found T cells differently infiltrated in distinct areas (Figure 1F). Moreover, we set a density of 0 as their cut‐offs to divide all patients to positive (Pos) group and negative (Neg) group.

**FIGURE 2:**
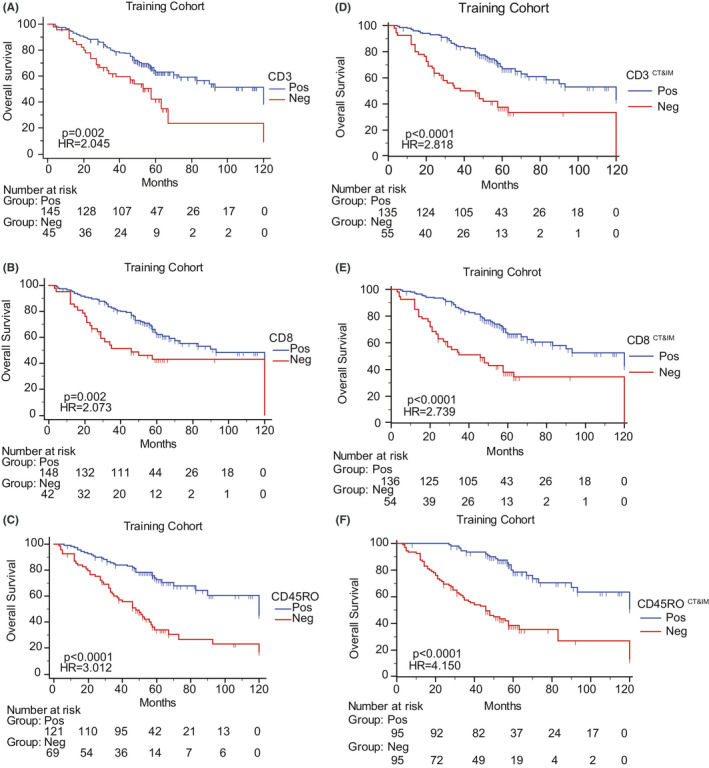
CD3, CD8, and CD45RO in mass and integrated CT and IM associated with OS. A‐C. Kaplan–Meier curves of CD3 (A), CD8 (B), CD45RO (C) in the training cohort. D‐F. Kaplan–Meier curves of CD3 CT&IM (D), CD8 CT&IM (E), CD45RO CT&IM (F) in the training cohort

We used Kaplan–Meier analysis to evaluate the relationship between CD3^+^, CD8^+^, and CD45RO^+^ T cells in all regions, and prognosis in the training cohort. The appearance of T cells was significantly correlated with favorable overall survival (OS) in mass and the three regions except for CD3^+^, CD8^+^ T cells in IN (Figure 2A‐C, CD3, *p* = 0.002 HR = 2.045; CD8, *p* = 0.002, HR = 2.073; CD45RO, *p* < 0.001, HR = 3.012, Figure S1A‐I, all *p* < 0.001 except CD3^IN^, *p* = 0.127). To improve efficiency, we incorporated T cells in regions that have significant correlations with OS (CD3^CT&IM^, CD8^CT&IM^, CD45RO^CT&IM^, and CD45RO^CT&IM&IN^). These curves showed infiltrating T cells in all regions associated with improved OS and strongly discriminate different prognosis with superior hazard risk between two groups (Figure 2D‐F, Figure S1J, all *p* < 0.001).

Hazard ratio (HR), C‐index, AUC, and AIC analyses were performed to compare the efficiency of T cells in mass and regions and we found these indexes of T cells combined with CT and IM were superior to than in mass and the three regions except for AUC of CD3^CT&IM^ (Table 1). Comparing with CD45RO^CT&IM&IN^, CD45RO^CT&IM^ had higher HR, C‐index, AUC, and lower AIC. Therefore, we would use CD3^CT&IM^, CD8^CT&IM^, and CD45RO^CT&IM^ as separate indicators in the following studies.

**TABLE 1:** Evaluation indexes of CD3, CD8, and CD45RO in training cohorts.

		Log‐rank test			C‐index	AUC	AIC
		p‐value^a^	HR	95%CI			
CD3	ALL	**0.002**	2.045	1.171, 3.571	0.570	0.618	755.535
	CT	**<0.0001**	2.564	1.591, 4.132	0.617	0.613	746.938
	IM	**<0.0001**	2.468	1.477, 4.123	0.609	0.654	748.431
	IN	0.127	1.399	0.892, 2.193			
	CT&IM	**<0.0001**	2.818	1.655, 4.799	0.631	0.635	746.022
CD8	ALL	**0.002**	2.073	1.159, 3.707	0.585	0.609	755.631
	CT	**0.0001**	2.248	1.378, 3.668	0.613	0.705	750.631
	IM	**0.0001**	2.267	1.380, 3.722	0.619	0.606	750.831
	IN	0.103	1.450	0.892, 2.358			
	CT&IM	**<0.0001**	2.739	1.603, 4.681	0.630	0.741	744.735
CD45RO	ALL	**<0.0001**	3.012	1.876, 4.837	0.644	0.634	739.482
	CT	**<0.0001**	3.020	1.954, 4.669	0.627	0.748	742.162
	IM	**<0.0001**	3.500	2.262, 5.415	0.645	0.764	735.633
	IN	**<0.0001**	2.427	1.559, 3.777	0.621	0.595	748.306
	CT&IM	**<0.0001**	4.150	2.649, 6.503	0.652	0.767	733.829
	CT&IM&IN	**<0.0001**	2.393	1.473, 3.888	0.644	0.643	739.483

Abbreviations: AIC, Akaike information criterionAUC, Area under curve; CI, Confidence interval; CT, The center of the tumor; HR, Hazard risk; IM, The invasive margin; IN, The interstitium.

^a^ p‐value <0.05 marked in bold font shows statistical significance.

### 3.2 Integrated evaluation of cytotoxic and memory T cells in quality and space well predicted overall survival

**FIGURE 3:**
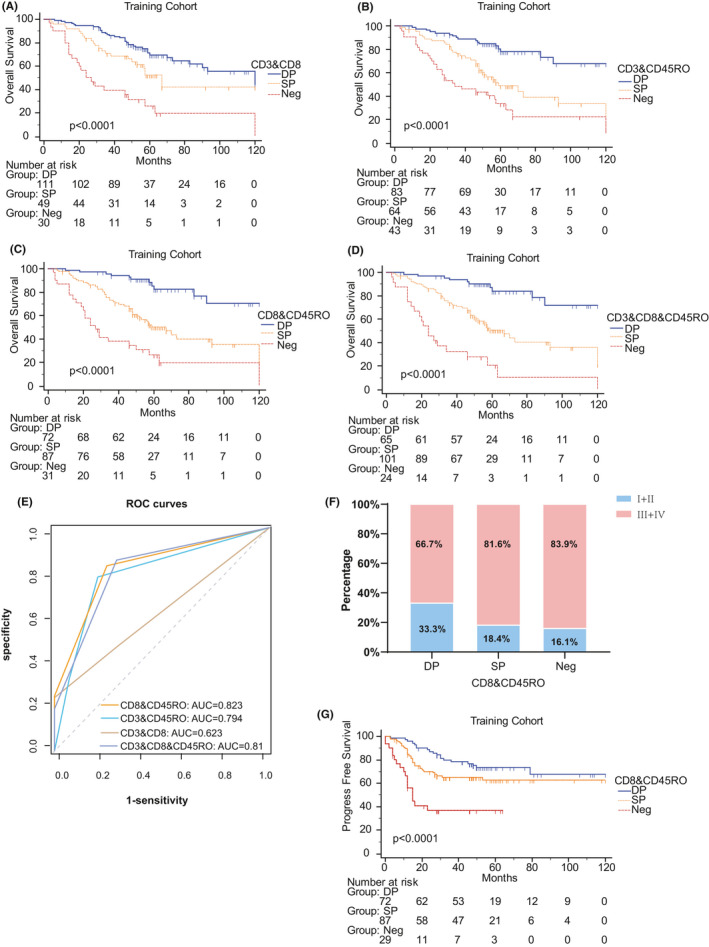
CD8&CD45RO is the best integrated indicator for prognosis among all combinations in the training cohort. (A‐D), Kaplan–Meier curves of combination CD3&CD8 (A), CD3&CD45RO (B), CD8&CD45RO, (C) and CD3&CD8&CD45RO (D) in term of OS. (E) The ROC curves of CD3&CD8, CD3&CD45RO, CD8&CD45RO, and CD3&CD8&CD45RO. (F) The distribution of FIGO stage in CD8&CD45RO groups. (G) Kaplan–Meier curve of CD8&CD45RO in term of PFS

We further investigated the combined forms of the three combined markers to search for the optimal results. First, we combined two of the three and all the three combined markers to identify patients with both positive markers as double positive (DP) group, patients with both negative as Neg group, and the others as single positive (SP) group based on the previous results. The survival curves manifested the patients in the DP groups of CD3&CD8, CD3&CD45RO, CD8&CD45RO, and CD3&CD8&CD45RO had the best prognosis, while the SP and Neg groups had the opposite (Figure 3A‐D, all *p* < 0.001). RMST was used to identify the prognostic significance of the SP group compared with DP and Neg groups with choosing the longest time as the end (Table 2). With the application of C‐index, AIC, RUC curves, and AUC, we detect that the integration of CD8^CT&IM^ and CD45RO^CT&IM^ had the strongest prediction effect than any single or integrated marker (Table 2, Figure 3E).

**TABLE 2:** Evaluation indexes of different combination of CD3, CD8, and CD45RO in training cohorts

	Log‐rank				RMST			C‐index	AUC	AIC
	p‐value		HR	95%CI	Value (months)	p‐value				
CD3&CD8	**<0.001**	Neg vs. DP	4.523	2.129, 9.606	DP (90.65) vs. SP (72.62)		**0.036**	0.664	0.623	736.274
		Neg vs. SP	1.810	1.079, 3.038	SP (72.62) vs. Neg (44.79)		**0.012**			
CD3&CD45RO	**<0.001**	Neg vs. DP	5.520	2.996,10.172	DP (98.91) vs. SP (72.15)		**<0.001**	0.689	0.794	729.659
		Neg vs. SP	3.055	1.891, 4.938	SP (72.15) vs. Neg (51.05)		**0.017**			
CD8&CD45RO	**<0.001**	Neg vs. DP	9.964	4.653, 21.338	DP (102.95) vs. SP (71.54)		**<0.001**	0.716	0.823	715.683
		Neg vs. SP	4.230	2.689, 6.653	SP (71.54) vs. Neg (44.74)		**0.004**			
CD3&CD8&CD45RO	**<0.001**	Neg vs. DP	12.047	4.962, 29.248	DP (103.74) vs. SP (71.98)		**<0.001**	0.704	0.81	716.696
		Neg vs. SP	4.226	2.698, 6.619	SP (71.98) vs. Neg (36.98)		**<0.001**			
IS	**<0.001**	Neg vs. DP	12.426	5.317, 29.039	DP (104.09) vs. SP (67.92)		**0.002**	0.753	0.854	707.294
		Neg vs. SP	5.167	3.259, 8.193	SP (67.92) vs. Neg (41.67)		**<0.001**			
FIGO stage	\	\	\	\	**\**		**\**	0.647	0.749	734.014

**Abbreviations:** AIC, Akaike information criterion; AUC, Area under curve; CI, Confidence interval; FIGO, The International Federation of Gynecology and ObstetricsHR, Hazard risk; IS, Immune score; RMST, Restricted mean time lost.

^a^ p‐value <0.05 marked in bold font shows statistical significance.

The correlation of clinicopathological characteristics with CD8&CD45RO was summarized in Table S2. In addition, we found the distribution of FIGO stage in CD8&CD45RO groups was extraordinarily unequal the Neg group had the most patients with late‐stage (Figure 3F). Moreover, a statistically significant interaction was observed for the predictive value of IS in term of progress free survival (PFS, *p* < 0.001).

### 3.3 Construction of the prognostic model incorporating immune infiltrates and clinical characteristic

**FIGURE 4:**
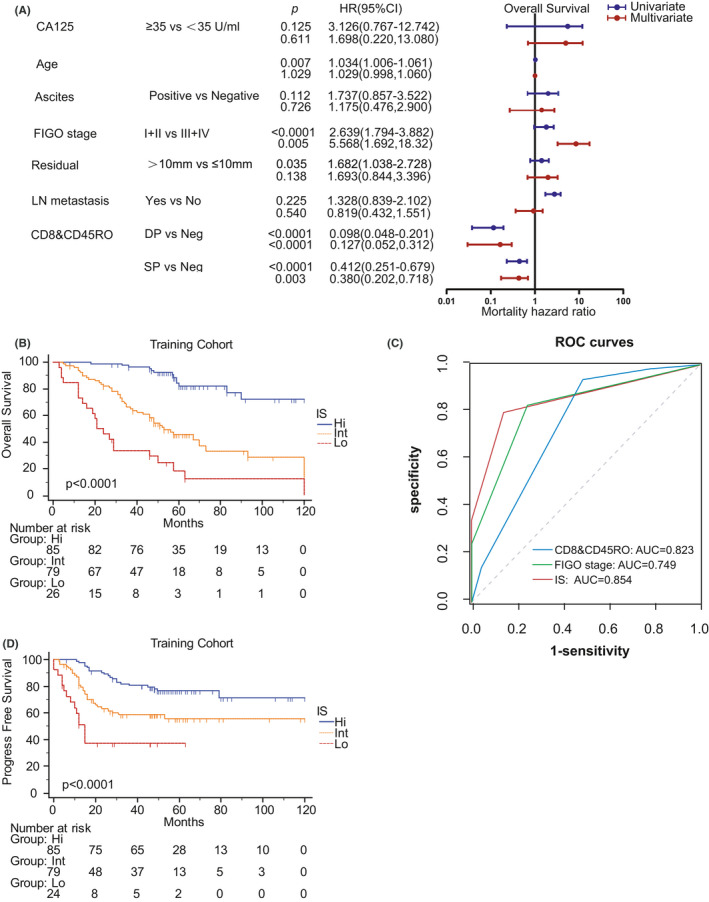
The IS consists of clinical characteristics and CD8&CD45RO in the training cohort. (A) The univariate and multivariate COX regression of clinical characteristics and CD8&CD45RO in term of OS. (B) The Kaplan–Meier curve of IS. (C) ROC curves of CD8&CD45RO, FIGO stage, and IS. (D) Kaplan‐Meier curve of IS in term of PFS

In the training cohort, the univariate COX regression analysis suggested that age, ascites, residual, lymph nodes (LN) metastasis, therapy response, FIGO stage, and CD8&CD45RO were statistically or nearly significant with overall survival and above all were analyzed by multivariate COX regression. The result of the multivariate COX regression indicated FIGO stage, and CD8&CD45RO were obviously associated with overall survival after backward stepwise selection (Figure 4A, Table S3). A prediction model named immunoscore (IS) was developed by incorporating FIGO stage and CD8&CD45RO to provide a quantitative tool for evaluating the survival probability rely on risk proportional regression. We set up FIGO stage I+II and CD8&CD45RO Neg as baseline, then the coefficients of the two variables were used to represent FIGO stage III+IV, CD8&CD45RO DP and SP. Finally, the sum of the value of FIGO stage and CD8&CD45RO was IS, and based this result, the IS was divided to three groups by X‐tile. The Kaplan–Meier curve and RMST indicated IS could stratify patients with HGSOC to three groups with different prognosis (Figure 4B, Table 2). Importantly, this model has the most remarkable effectiveness than the indicator of CD8&CD45RO and FIGO stage by comparison of ROC, C‐index, AUC, and AIC (Figure 4C, Table 3). IS was also observed significantly associated with PFS but the efficiency of CD8&CD45RO and IS were both weaker than FIGO (Figure 4D, Table 3).

**TABLE 3:** Evaluation indexes of CD&CD45RO, IS, and FIGO in training cohort

		Log‐rank				RMST			C‐index	AUC	AIC
		p‐value^a^		HR	95%CI	Value (months)	p‐value^a^				
OS	CD8&CD45RO	**<0.0001**	Neg vs. DP	9.964	4.653–21.338	DP (102.849, 93.751–111.963) vs. SP (75.262, 59.920–90.603)		**<0.0001**	0.716	0.823	715.683
			Neg vs. SP	4.230	2.689–6.653	SP (75.262, 59.920–90.603) vs. Neg (48.678, 38.824–58.536)		**0.004**			
						DP (102.849, 93.751–111.963) vs. Neg (48.678, 38.824–58.536)		**<0.0001**			
	IS	**<0.0001**	Lo vs. Hi	12.426	5.317–29.039	Hi (104.091, 96.312–111.872) vs. Int (82.658, 68.065–97.242)		**0.002**	0.753	0.854	707.294
			Lo vs. Int	5.167	3.259–8.193	Int (82.658, 68.065–97.242) vs. Lo (55.080, 44.680–65.477)		**0.003**			
						Hi (104.091, 96.312–111.872) vs. Lo (55.080, 44.680–65.477)		**<0.0001**			
	FIGO stage	\	\	\	\	**\**		**\**	0.647	0.749	734.014
PFS	CD8&CD45RO	**<0.0001**	Neg vs. DP	4.152	1.804–9.554	DP (92.793, 82.212–103.362) vs. SP (81.741, 70.749–92.740)		0.156	0.554	0.562	660.364
			Neg vs. SP	1.547	0.932–2.560	SP (46.560, 41.450–51.671) vs. Neg (29.808, 19.973–39.651)		**0.003**			
						DP (53.986, 49.751–58.212) vs. Neg (29.808, 19.973–39.651)		**<0.0001**			
	IS	**<0.0001**	Lo vs. Hi	5.271	2.087–13.308	Hi (95.977, 86.661–105.305) vs. Int (74.382, 62.300–86.463)		**0.006**	0.507	0.504	664.346
			Lo vs. Int	2.217	1.338–3.674	Int (42.575, 37.085–48.080) vs. Lo (28.571, 17.713–39.420)		**0.024**			
						Hi (54.490, 50.948–58.034) vs. Lo (28.571, 17.713–39.420)		**<0.0001**			
	FIGO stage	\	\	\	\	**\**		**\**	0.614	0.619	651.603

**Abbreviations:** AIC, Akaike information criterionAUC, Area under curve; CI, Confidence interval; FIGO, The International Federation of Gynecology and Obstetrics; HR, Hazard risk; IS, Immune score; RMST, Restricted mean time lost.

^a^ p‐value <0.05 marked in bold font shows statistical significance.

**FIGURE 5:**
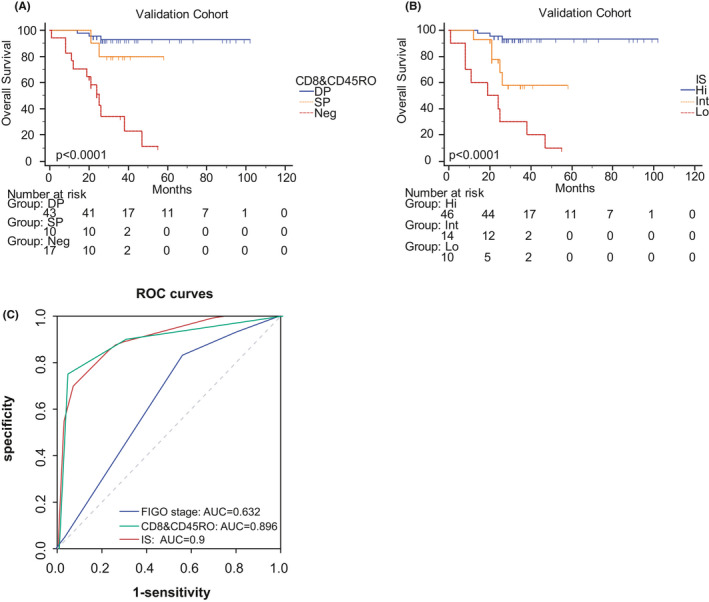
The validation of CD8&CD45RO and IS in the validation cohort. (A) Kaplan–Meier curve of CD8&CD45RO in term of OS. (B) Kaplan–Meier curve of IS in term of OS. (C) ROC curves of CD8&CD45RO, FIGO stage, and IS

CD8&CD45RO was further verified by Kaplan–Meier in the validation cohort which showed the same statistical significance as the training cohort (Figure 5A). Moreover, we also calculated IS and performed its Kaplan–Meier curve (Figure 5B). It can be confirmed that the model is superior to CD8 & CD45RO and FIGO stage in prediction efficiency by calculating ROC, C‐index, AUC, and AIC (Figure 5C, Table 4). There were some differences between the results of the two cohorts which may be due to the limited amount of the validation cohort.

**TABLE 4:** Evaluation indexes of different combination of CD3, CD8, and CD45RO in validation cohort

	Log‐rank				RMST		C‐index	AUC	AIC
	p‐value^a^	HR		95%CI	Value (months)	p‐value^a^			
CD8&CD45RO	**<0.0001**	Neg vs. DP	16.805	4.55–62.062	DP (55.31, 52.36–58.26) vs. SP (51.00, 42.31–59.70)	0.358	0.796	0.896	110.542
		Neg vs. SP	3.113	0.795–12.179	SP (48.60, 40.65–56.55) vs. Neg (26.76,18.41–35.11)	**<0.0001**			
					DP (52.52, 49.80–55.25) vs. Neg (26.76, 18.41–35.11)	**<0.0001**			
IS	**<0.0001**	Lo vs. Hi	23.055	4.495–118.2547	Hi (55.49, 52.72–58.25) vs. Int (42.70, 32.27–53.13)	**0.020**	0.811	0.900	109.446
		Lo vs. Int	7.189	2.001–25.822	Int (40.960, 31.378–50.542) vs. Lo (23.60,13.008–34.192)	**0.017**			
					Hi (52.69, 50.14–55.24) vs. Lo (23.60,13.01–34.19)	**<0.0001**			
FIGO stage	\	\	\	\	**\**	**\**	0.598	0.632	134.850

**Abbreviations:** AIC, Akaike information criterion; AUC, Area under curve; CI, Confidence interval; FIGO, The International Federation of Gynecology and ObstetricsHR, Hazard risk; IS, Immune score; RMST, Restricted mean time lost.

^a^ p‐value <0.05 marked in bold font shows statistical significance.

## 4 DISCUSSION

Efficient assessment of patients’ risk after the surgery is crucial for the therapy of HGSOC.^24^, ^25^ In this study, the heterogeneity of immune infiltrates was observed in HGSOC based on CD3^+^ T cells, CD8^+^ cytotoxic T cells, and CD45RO^+^ memory T cells. We had evaluated the predictive effect of CD3^+^, CD8^+^, and CD45RO^+^ T cells in CT, IM, and IN regions and various kinds of combinations in the training cohort. The combinations of T cells infiltration in regions had more powerful predictive efficiency than those in mass. Furthermore, we got a prominent integrated indicator, CD8&CD45RO which could divide patients with epithelial ovarian cancer to three groups of different risk through different combinations and verification. Lastly, a quantitative tool was constructed to evaluate the survival probability depend on clinical characteristics and CD8&CD45RO which had been verified in the validation cohort.

Comparing with the other joint indicators, CD8&CD45RO appeared the most efficient in predicting survival outcomes after surgery which could be applied in patients with different pathological diagnoses. According to our findings, patients can be stratified into three prognostic categories with significantly different overall survival. The Hi group had a prolonged OS differed from that of patients in the Lo group, as well as favorable clinical characteristics. Multivariate COX analysis, combining age, ascites, residual, lymph nodes (LN) metastasis, therapy response, FIGO stage, and CD8&CD45RO, supports the advantage of CD8&CD45RO over HGSOC in predicting survival which indicated the possibility of application in clinical practice. Besides, the IS comprising FIGO stage and LVSI exhibited more robust performance in predicting OS probability instead of PFS. These observations possessed relatively high credibility and applicability on account of clinical samples. Unfortunately, the size of the population was limited, and our research lacked the exploration of mechanisms.

In general, the tumor‐infiltrating T cells play important roles in epithelial ovarian cancer, including cytotoxic T cells, memory T cells, regulatory T cells, and so on.^26^ In our research, we focused on cytotoxic T cells and memory T cells which have not been jointly reported in HGSOC. CD3^+^, CD8^+^, CD45RO^+^ cells represent T cells, cytotoxic T cells, and memory T cells. Although CD3^+^ was not included in final model, the infiltration of CD3^+^ T cells in tumor was obviously associated with prognosis. CD8^+^ cytotoxic and CD45RO^+^ memory T cells play vital roles in antitumor immune responses and could supply more new therapeutic targets.^27^, ^28^, ^29^, ^30^, ^31^, ^32^, ^33^ It has been confirmed that high levels of tumor‐infiltrating cytotoxic T cells and memory T cells associated with favorable survival and clinical characteristics in many human cancers which suggested a protective role of immune infiltrates of T cells in previous studies.^34^, ^35^, ^36^, ^37^, ^38^, ^39^, ^40^ Previous clinical trials with immune checkpoint inhibitors have failed in terms of clinical outcome for ovarian cancer which suggested the multiple immune models and mechanisms. In our research, Cytotoxic CD8^+^ T cell in memory phase may have important function in ovarian cancer TME which could supply new a new approach for the study of immune therapy. A variable description of subjective ratings and incomplete combinations favors the previously found insufficient reliability.^13^, ^17^ Our explorations examined all combinations of the immune infiltrates’ density and the location of immune cell populations in distinct tumor territories. We will explore other T cells such as CD4 T cell, type 1 T cell, regulatory T cell and so on in next study on tumor‐infiltrating T cells in HGSOC.

HR of log‐rank in Kaplan–Meier analysis can only indicate the risk of death which is not a probability measure and the p‐value can not prove the significance between the intermediate group and the other groups associated with OS.^41^, ^42^ When using HR, it is necessary to assume proportional hazards (PH), that is, HR will not change with time, and the failure of proportional hazards assumption will often occur during the long follow‐up.^41^, ^42^ Without a reference value for hazard in the low group, it is not easy for clinical practitioners and patients to understand the implications of HR values.^43^ Therefore, we used RMST as a quantitative measure to solve the problems above. For IS in the training cohort, we evaluate the RMSTs over 120 months of follow‐up and obtained RMST values of 104.09 months in the high group, 67.92 months in the intermediate group, and 41.67 months in the low group. The RMST difference is 36.17 months (*p* < 0.001) between patients in high group and those in intermediate group, 26.25 months (*p* < 0.001) between patients in intermediate group and those in low group, and 62.41 months (*p* < 0.001) between patients in the high group and in the low group. RMST supplied a clinically meaningful summary measure of survival time for each group.^40^, ^41^ Moreover, C‐index and AUC were performed to evaluate the prediction efficiency of all markers for survival time and AIC was also used to measure the goodness of statistical model fitting.^44^ IS was the most efficient and robust prognostic marker for patients with HGSOC based multiple indexes mentioned.

In summary, our investigations supply powerful evidence for the usefulness of the combined evaluation of cytotoxic and memory T cells for the prediction of survival in patients with epithelial cancer. To be incorporated into clinical practice, we developed a new predictive model that included clinical features and immune infiltrates to enhance the credible impact on the outcome.

## CONFLICT OF INTEREST

The authors declare no conflict of interest.

## ETHICAL APPROVAL AND ETHICAL STANDARDS

This study was conducted following the Declaration of Helsinki and was approved by the Ethics Committee of Obstetrics & Gynecology Hospital, Fudan University (Kyy2016‐49, for training cohort) and the Ethics Committee of Suzhou Municipal Hospital (2008–7–15, for validation cohort).

## Supporting information

Fig S1

Table S1‐S3

## Data Availability

The data that support the findings of this study are available from the corresponding author upon reasonable request.
