# Supplementary material for: Spatial cytotoxic and memory T cells in tumor predict superior survival outcomes in patients with high‐grade serous ovarian cancer
Source: Cancer Med. 2021 May 5;10(12):3905–18. doi: 10.1002/cam4.3942 (PMC8209602; doi:10.1002/cam4.3942)
Supplement: Supplementary file 1 — Fig S1 [file CAM4-10-3905-s001.pdf]

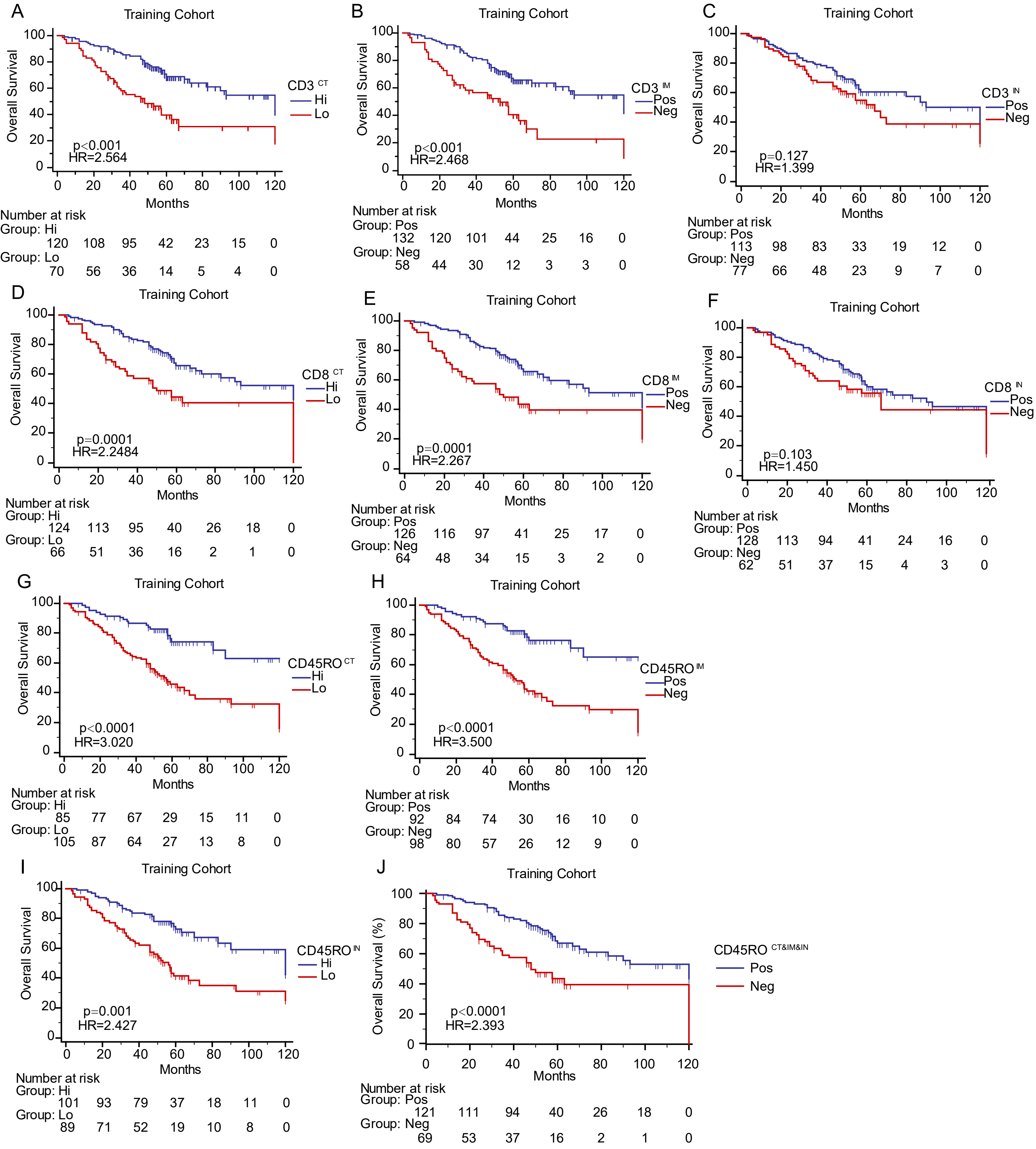

Fig. S1 CD3, CD8, and CD45RO in CT, IM, and IN associated with OS.
A-C. Kaplan-Meier curves of CD3 CT (A), CD3 IM (B), and CD3 IN (C) in the training cohort.
D-F. Kaplan-Meier curves of CD8 CT (D), CD8 IM (E), and CD8 IN (F) in the training cohort.
G-J. Kaplan-Meier curves of CD45RO CT (G), CD45RO IM (H), CD45RO IN (I), and CD45RO CT&IM&IN (J) in the training cohort.
