# Supplementary material for: Spatial cytotoxic and memory T cells in tumor predict superior survival outcomes in patients with high‐grade serous ovarian cancer
Source: Cancer Med. 2021 May 5;10(12):3905–18. doi: 10.1002/cam4.3942 (PMC8209602; doi:10.1002/cam4.3942)
Supplement: Supplementary file 2 — Table S1‐S3 [file CAM4-10-3905-s002.docx]

| **Table S1. Clinicopathological characteristics of training and validation cohorts.** | | | | | | | |
| --- | --- | --- | --- | --- | --- | --- | --- |
| **Group** | **Pooled(n=277)** | | **Training cohort（n=190）** | | **Validation cohort(n=70)** | | **p-value^a^** |
|  | ***No.*** | **%** | ***No.*** | **%** | ***No.*** | **%** |  |
| **Age (years)** | 56.0±10.4 | | 57.4±10.2 | | 53.5±10.5 | | 0.678 |
| **Ascites** |  |  |  |  |  |  | **0.007** |
| ≤20ml | 56 | 21.5% | 32 | 16.8% | 24 | 34.3% |  |
| >20ml | 166 | 63.8% | 133 | 70.0% | 33 | 47.1% |  |
| Unknown | 38 | 14.6% | 25 | 13.2% | 13 | 18.6% |  |
| **CA125** |  |  |  |  |  |  | 0.856 |
| ≤35mg/L | 16 | 6.2% | 12 | 6.3% | 4 | 5.7% |  |
| >35mg/L | 226 | 86.9% | 174 | 91.6% | 52 | 74.3% |  |
| Unknown | 18 | 6.9% | 4 | 2.1% | 14 | 20.0% |  |
| **Residual** |  |  |  |  |  |  | 0.478 |
| ≤10 mm | 171 | 65.8% | 148 | 77.9% | 23 | 32.9% |  |
| >10mm | 58 | 22.3% | 48 | 21.6% | 10 | 14.3% |  |
| Unknown | 48 | 18.5% | 1 | 0.5% | 47 | 67.1% |  |
| **LN Metastasis** |  |  |  |  |  |  | 0.110 |
| Yes | 107 | 41.2% | 90 | 47.3% | 17 | 24.3% |  |
| No | 122 | 46.9% | 94 | 49.5% | 28 | 40.0% |  |
| Unknown | 41 | 15.8% | 6 | 3.2% | 35 | 50.0% |  |
| **Therapy response** |  |  |  |  |  |  | ***** |
| CR+PR | 162 | 62.3% | 152 | 80% | 10 | 14.3% |  |
| PD+SD | 27 | 10.4% | 16 | 8.4% | 11 | 15.7% |  |
| Unknown | 71 | 27.3% | 22 | 11.6% | 49 | 70.0% |  |
| **FIGO stage** |  |  |  |  |  |  | 0.105 |
| I | 29 | 11.2% | 18 | 9.5% | 11 | 15.7% |  |
| II | 38 | 14.6% | 24 | 14.2% | 14 | 20.0% |  |
| III | 167 | 64.2% | 125 | 65.8% | 42 | 60.0% |  |
| IV | 23 | 8.8% | 20 | 10.5% | 3 | 4.3% |  |

^a^ p-value < 0.05 marked in bold font shows statistical significance.

* Too much missing data to calculate p-value

**Abbreviations:**

LN Lymph node

CR Complete response

PR Partial response

PD Progressive disease

SD Stable disease

FIGO The International Federation of Gynecology and Obstetrics

**Table S2. Correlation of clinicopathological characteristics with CD8&CD45RO in training and validation cohorts.**

| **Characteristics** |  | **Training cohort** | | | |  | | **Validation cohort** | | |
| --- | --- | --- | --- | --- | --- | --- | --- | --- | --- | --- |
|  | **Low** | **Intermediate** | **High** | ***P*-value ^a^** | **Low** | | **Intermediate** | | **High** | **p-value ^a^** |
| **All patients** | 72 | 87 | 31 |  | 17 | | 10 | | 43 |  |
| **Age (years)** | 55.0±10.6 | 58.5±10.2 | 58.0±9.3 | 0.1672 | 55.0±12.8 | | 53.2±11.4 | | 54.7±9.5 | 0.971 |
| **Ascites**  ≤20ml  >20ml  Unknown | 16  46  10 | 12  65  15 | 4  27  0 | 0.246 | 6  9  2 | | 7  3  0 | | 11  21  11 | 0.135 |
| **CA125 (U/ml)**  ≤35  ＞35  Unknown | 7  63  2 | 5  80  2 | 0  31  0 | 0.162 | 2  11  4 | | 0  10  0 | | 2  31  10 | 0.340 |
| **Residual** |  |  |  | 0.470 |  | |  | |  | 0.871 |
| ≤10mm  ＞10mm  Unknown | 58  14  0 | 67  19  1 | 23  8  0 |  | 3  8  6 | | 2  3  5 | | 5  12  26 |  |
| **LN Metastasis** |  |  |  | 0.992 |  | |  | |  | 0.516 |
| Yes | 33 | 42 | 15 |  | 7 | | 6 | | 15 |  |
| No | 35 | 43 | 16 |  | 3 | | 2 | | 12 |  |
| Unknown | 4 | 2 | 0 |  | 7 | | 2 | | 16 |  |
| **Therapeutic efficacy** |  |  |  | 0.585 |  | |  | |  | * |
| CR+PR | 60 | 68 | 24 |  | 6 | | 1 | | 4 |  |
| PD+SD | 7 | 6 | 3 |  | 1 | | 2 | | 7 |  |
| Unknown | 5 | 13 | 4 |  | 10 | | 7 | | 32 |  |
| **FIGO stage** |  |  |  | 0.121 |  | |  | |  | 0.245 |
| I  II  III  IV | 9  15  45  3 | 6  10  58  13 | 3  2  22  4 |  | 5  2  9  1 | | 3  1  6  0 | | 3  11  27  2 |  |

^a^ p-value < 0.05 marked in bold font shows statistical significance.

* Too much missing data to calculate p-value

**Abbreviations:**

LN Lymph node

CR Complete response

PR Partial response

PD Progressive disease

SD Stable disease

FIGO The International Federation of Gynecology and Obstetrics

**Table S3. Univariate and multivariate COX regression of clinicopathological characteristics and CD8&CD45RO in training cohort.**

^a^ p-value < 0.05 marked in bold font shows statistical significance.

|  | | **OS** | | | | | | | | |
| --- | --- | --- | --- | --- | --- | --- | --- | --- | --- | --- |
|  |  | **Univariate** | | | | **Multivariate** | | | | |
|  |  | **p-value^a^** | | | **HR(95%CI)** | **p-value^a^** | | | **HR(95%CI)** | |
| **Age** | | | **0.007** | 1.035(1.009,1.061) | | | 0.068 | 1.029(0.998,1.060) | |  |
| **Ascites** | | | 0.125 | 1.7374(0.857,3.522) | | | 0.726 | 1.175(0.476,2.900) | |  |
| **CA125** | | | 0.112 | 3.126(0.767,12.743) | | | 0.611 | 1.698(0.220,13.080) | |  |
| **Residual** | | | **0.035** | 1.682(1.038,2.728) | | | 0.138 | 1.693(0.844,3.396) | |  |
| **LN Metastasis** | | | 0.225 | 1.328(0.839,2.102) | | | 0.540 | 0.819(0.432,1.551) | |  |
| **FIGO stage** | | | **<0.001** | 2.639(1.794,3.882) | | | **0.005** | 5.568(1.692,18.32) | |  |
| **CD8&CD45RO** | **DP vs Neg** | | **<0.0001** | 0.098(0.048,0.201) | | | **<0.0001** | 0.127(0.052,0.312) | |  |
|  | **SP vs Neg** | | **<0.0001** | 0.412(0.251,0.679) | | | **0.003** | 0.380(0.202,0.718) | |  |

**Abbreviations:**

HR Hazard risk

CI Confidence interval

OS Overall survival

LN Lymph node

FIGO The International Federation of Gynecology and Obstetrics
